# Production of Vitamin B_12_ in *Escherichia coli* Using a Thermal Switch to Control Pathway Genes

**DOI:** 10.4014/jmb.2412.12068

**Published:** 2025-04-11

**Authors:** Kaize Kong, Feitao Chen, Huan Fang, Pingtao Jiang, Xinfang Zhao, Jijiao Zhang, Huina Dong, Hongxing Jin, Dawei Zhang

**Affiliations:** 1 School of Chemical Engineering and Technology, Hebei University of Technology, Tianjin, P.R. China; 2 Tianjin Institute of Industrial Biotechnology, Chinese Academy of Sciences, Tianjin, P.R. China; 3 College of Biotechnology, Tianjin University of Science & Technology, Tianjin, P.R. China; 4 State Key Laboratory of Engineering Biology for Low-Carbon Manufacturing, Tianjin Institute of Industrial Biotechnology, Chinese Academy of Sciences, Tianjin, P.R. China; 5 University of Chinese Academy of Sciences, Beijing, P.R. China; 6 School of Food Science, Dalian University of Technology, Dalian, P.R. China

**Keywords:** *cI857* Repressor, *Escherichia coli*, *lacI*, thermal switch, vitamin B_12_

## Abstract

Isopropyl β-D-thiogalactopyranoside (IPTG), while widely utilized for inducing gene expression in systems governed by T7*lac* and related promoters, poses significant challenges due to its toxicity and expense, prompting the exploration of alternative induction strategies. In this study, we developed a series of inducer-free vitamin B_12_-producing strains featuring thermally regulated pathway genes. We engineered a thermal switch by replacing the *lacI* promoter with the P_R_ promoter, which is regulated by the temperature-sensitive repressor cI857 from the λ bacteriophage. As a result, target genes driven by T7*lac* or other *lac*-derived promoters containing *lac* operators were expressed upon lowering the temperature. Our findings indicate that culturing at 37°C and then shifting to 32°C when the optical density at 600 nm reaches 2 is the most effective strategy for vitamin B_12_ production. Additionally, the vitamin B12 titer increased by 37.96% after introducing an *ssrA* degradation tag at the C-terminus of *lacI*. This study introduces a novel strategy for vitamin B_12_ production that circumvents the need for IPTG by implementing a thermal switch. This approach may have significant implications for chemical bioproduction processes that have traditionally relied on IPTG for gene induction.

## Introduction

Vitamin B_12_ is the most complex vitamin in terms of its structure and synthesis pathway. In nature, its synthesis primarily takes place in microorganisms, with subsequent accumulation in animals through the food chain. The active forms of vitamin B_12_ include adenosylcobalamin and methylcobalamin. As an essential coenzyme, vitamin B_12_ plays a critical role in human metabolism, particularly in DNA synthesis, erythrocyte formation, and the maintenance of nerve function [1]. Despite its significance in physiological processes, traditional methods for vitamin B_12_ production are often characterized by high costs and low efficiency, necessitating the exploration of more effective and economical production techniques by the scientific community. The rapid advancements in synthetic biology and metabolic engineering have shifted attention towards utilizing microbial cell factories for the production of high-value compounds [2]. *Escherichia coli* is frequently employed as a model organism in metabolic engineering due to its well-characterized genome, rapid growth rate, and ease of genetic manipulation. Recently, we developed 14 antibiotic-free, plasmid-free producers by integrating vitamin B_12_ pathway genes under the regulation of the T7*lac* and tac promoters into the *E. coli* chromosome [3]. The *lac* operon system, a widely used gene expression regulation mechanism in bacteria, comprises a *lac* promoter, an operator sequence (*lacO*), and structural genes (*lacZ*, *lacY*, *lacA*), which can be induced through the addition of IPTG [4, 5]. The T7*lac* promoter is a hybrid that combines elements from the T7 bacteriophage promoter with *lacO*, and it is also activated by IPTG. However, the induction of the T7 system with IPTG facilitates rapid protein overexpression, which can overwhelm cellular folding mechanisms and result in the aggregation of misfolded proteins [6, 7]. Studies have indicated that IPTG can heighten cellular sensitivity to various toxic substances, potentially leading to damage in host cells [8-11].

Temperature-sensitive promoters, such as the P_R_ and P_L_ promoters, enable precise regulation of gene expression across varying temperature conditions [12]. The low-temperature induction of *cI857* represents an effective strategy for regulating gene expression, particularly in the production of recombinant proteins [13, 14]. The cI857 protein, a temperature-sensitive repressor in the l phage, operates in conjunction with the P_L_ and P_R_ promoters. These promoters inhibit the transcription of downstream genes when the cI857 repressor binds. Specifically, at temperatures below 37°C, the cI857 repressor tightly associates with the promoter, preventing RNA polymerase from binding and thus inhibiting gene transcription. Conversely, when the temperature exceeds 37°C, the cI857 repressor becomes less stable and dissociates from the promoter, allowing RNA polymerase to bind and initiate the transcription of the target gene. This temperature-induced mechanism not only significantly enhances the yield of recombinant proteins but also minimizes issues related to non-specific expression and protein misfolding that may occur at lower temperatures. Furthermore, the design of a thermoinducible T7*lac* promoter circumvents the toxicity associated with IPTG and enhances the flexibility of gene expression [15]. Although several studies have investigated the expression of the T7 RNA polymerase gene under the heat-inducible l P_L_ promoter to boost recombinant protein yields [16-18], elevated temperatures ranging from 37°C to 42°C are detrimental to vitamin B_12_ production, while 32°C is identified as the optimal temperature due to the expression of 28 heterologous genes involved in the vitamin B_12_ biosynthetic pathway [19]. Recently, a low-temperature inducible system for activating the T7*lac* promoter has been developed [20]. However, the evaluation of this system was conducted in a Luria−Bertani (LB) medium. Previous studies have shown that the presence of lactose in yeast extract promotes the autoinduction of the lacUV5 promoter [21], which complicates the assessment of the precise control afforded by the low-temperature inducible system. Furthermore, it remains to be investigated whether this system can be applied to other promoters.

In this study, we aimed to regulate the genes involved in the vitamin B_12_ synthesis pathway driven by the T7*lac* promoter using a thermal switch in *E. coli*, thereby replacing IPTG induction. We evaluated this system using recombinant strains cultured in minimal media to eliminate the confounding effects of lactose. This approach allowed for clearer insights into the direct impact of temperature on promoter activity. We achieved this by expressing the *lacI* gene via the P_R_ or tandem P_R_ and P_L_ promoters, under the control of the thermolabile mutant repressor cI857, allowing the transcription of the T7*lac* promoter and other *lac*-derived promoters containing *lac* operators to be controlled by temperature. Subsequently, this thermal switch was utilized to manipulate the genes involved in the vitamin B_12_ biosynthetic pathway, ultimately identifying optimal conditions for enhancing vitamin B_12_ production. This research offers novel insights for the industrial production of vitamin B_12_ and establishes a foundation for the future synthesis of other high-value compounds through thermal switches for pathway gene regulation.

## Materials and Methods

### Materials

Vitamin B_12_ (purity >99%) was obtained from Aladdin Scientific. For routine polymerase chain reaction (PCR) experiments, Q5 High-Fidelity DNA Polymerase from New England Biolabs (USA) was utilized. In colony PCR experiments, we employed 2× SuperTaq PCR StarMix (Dye) reagents provided by Genstar (China). Additionally, other molecular cloning reagents, such as restriction endonuclease, T4 DNA ligase, and T4 polynucleotide kinase, were procured from Thermo Fisher Scientific (USA). The ClonExpress Ultra One-Step Cloning Kit V2 was acquired from Vazyme (China). The tandem P_R_ and P_L_ promoters were synthesized by GENEWIZ (China) to serve as templates for gene cloning.

### Bacterial Strains and Plasmids

The bacterial strains and plasmids utilized in this study are specified in Table S1, while the primers employed for amplification are detailed in Table S2. The *endA* gene in MG1655 (DE3) was successfully knocked out using CRISPR/Cas9 technology [22], resulting in the strain designated as FH224. The superfolder green fluorescent protein (*sfGFP*) gene was cloned into the *XbaI* site of pCas9-*hsdR* using the ClonExpress Ultra One Step Cloning Kit, producing the construct pCas9-*hsdR*-*sfGFP*. This plasmid was amplified with inverse primers; the resulting gene fragments were phosphorylated using T4 polynucleotide kinase and subsequently re-circularized, yielding the plasmids pCas9-*hsdR*-lac-*sfGFP*, pCas9-*hsdR*-tac-*sfGFP*, pCas9-*hsdR*-trc-*sfGFP*, and pCas9-*hsdR*-lacUV5-*sfGFP*. Following this, the *sfGFP* gene, driven by the T7*lac* promoter, was integrated into the genome of FH224 via CRISPR/Cas9 [22], generating the strain FH663. Further genetic modifications were made to the *lacI* gene using the CRISPR/Cas9 system [22].

The plasmid backbone of pRed_Cas9 was divided into two segments and amplified with the primer pairs Pcas9-1-F-Pr-lacI/Pcas9-1-R-Pr-lacI and Pcas9-2-F-Pr-lacI/Pcas9-2-R-Pr-lacI, followed by digestion with *DpnI* to eliminate the plasmid template. The upstream and downstream homology arms of the *lacI* gene were amplified from the *E. coli* MG1655 genome, using the primers Pr-lacI-up-F/Pr-lacI-up-R and Pr-lacI-dn-F/Pr-lacI-dn-R, respectively. The cI857 gene and P_R_ promoter were amplified from the pCP20 plasmid and fused into a single fragment with the upstream and downstream homology arms using overlapping extension PCR with the primers PR-lacI-up-F and PR-lacI-dn-R. Subsequently, the plasmid backbones, upstream homology arm, *cI857* gene, P_R_ promoter, and downstream homology arm were assembled using the ClonExpress Ultra One Step Cloning Kit. Additional plasmids, including pCas9-RBS-lacI, pCas9-PRPL, pCas9-lacI-LVA tag, pCas9-lacI-LAA tag, and pCas9-lacI-ASV tag, were constructed in a similar fashion. These plasmids were transformed into the respective host strains for genome editing, resulting in the recombinant strains listed in Table S1.

### Medium and Growth Conditions

Bacterial cultures from frozen storage tubes were streaked onto LB solid medium supplemented with the appropriate antibiotics and incubated at 37°C for 12 to 14 h. A single colony was subsequently selected and inoculated into 5 ml of LB liquid medium, which was then incubated at 37°C overnight with shaking at 200 rpm. The overnight cultures were transferred into 250 ml sterile conical flasks containing 30 ml of modified M9 minimal (MM9) medium, composed of 6.78 g/l Na_2_HPO_4_, 3.0 g/l KH_2_PO_4_, 0.5 g/l NaCl, 2.0 g/l NH_4_Cl, 1.0 g/l (NH_4_)_2_SO_4_, 1 mM MgSO_4_, 0.1 mM CaCl_2_, 20 g/l glycerol, with an initial optical density at 600 nm (OD_600_) of 0.1. The cultures were then incubated at 37°C with shaking at 200 rpm. Once the OD_600_ reached 0.8, the temperature was reduced to 32°C to induce *sfGFP* expression, and 1 mM IPTG was added to the control strain FH663. After induction, the cultures continued to be incubated at 37°C with shaking at 200 rpm for 48 h. Three biological replicates were conducted for each experimental group. After fermentation, an appropriate volume of the fermentation broth was collected, and cells were harvested by centrifugation and washed twice with PBS. The cells were then resuspended in PBS, adjusting the OD_600_ to approximately 0.2. The fluorescence intensity of the resuspended cell culture was measured using a Synergy Neo2 multifunctional microplate reader (BioTek, USA), with the fluorescence intensity of the green fluorescent protein (GFP) recorded using an excitation wavelength of 485 nm and an emission wavelength of 525 nm. The OD_600_ of the cells was assessed using a spectrophotometer (AOELAB UV-1000, China). The measured fluorescence intensity was normalized to the OD_600_ to calculate the specific fluorescence.

For flask cultivation of vitamin B_12_ production, overnight cultures of recombinant *E. coli* strains were inoculated into 250 ml Erlenmeyer flasks containing 30 ml of MR01 medium, with an initial OD_600_ of 0.05. The MR01 medium (pH 7.0) consists of 20 g/l glycerol, 6.67 g/l KH_2_PO_4_, 4 g/l (NH_4_)_2_HPO_4_, 0.8 g/l MgSO_4_·7H_2_O, 0.8 g/l citric acid, 20 mg/l CoCl_2_·6H_2_O, 90 mg/l 5,6-dimethylbenzimidazole (DMBI), 2 g/l glycine, 10 g/l succinic acid, 0.5 g/l betaine, and 0.5% trace metal solution (V/V). The trace metal solution included (per liter) the following components: 10 g FeSO_4_·7H_2_O, 2 g CaCl_2_, 2.2 g ZnSO_4_·7H_2_O, 0.5 g MnSO_4_·4H_2_O, 1 g CuSO_4_·5H_2_O, 0.1 g (NH_4_)_6_Mo_7_O_24_·4H_2_O, and 0.02 g Na_2_B_4_O_7_·10H_2_O, in a 0.5 M HCl solution. Gene expression was induced by shifting the temperature to 32°C.

### Real-Time Online Monitoring of *sfGFP* in the BioLector Microbioreactor

Fresh single colonies were selected from the activation plate, inoculated in 5 ml of LB liquid medium, and incubated at 37°C with shaking at 200 rpm for 12-15 h to obtain overnight cultures. The overnight culture was transferred to a sterile 10 ml centrifuge tube and centrifuged at 4,000 rpm for 10 min. The supernatant was discarded, and the bacterial pellet was retained. The bacteria were resuspended in 5 ml of sterile water, and the centrifugation step was repeated three times to eliminate residual medium. Subsequently, 5 ml of fresh fermentation medium was added to resuspend cells, and a 10-fold dilution was performed to determine the initial OD_600_ value of the seed solution. The seed solution was inoculated into a sterile 10 ml centrifuge tube containing 5 ml of the corresponding fermentation medium, achieving an initial OD_600_ of 0.05. Following thorough mixing, 900 ml aliquots from each tube were transferred into a 48-well FlowerPlate. After sealing the plate, it was placed in a BioLector XT micro bioreactor and incubated at 37°C with agitation set at 1,400 rpm. When the bacterial biomass reached an OD_600_ of approximately 0.8, isopropyl b-D-1-thiogalactopyranoside (IPTG) was introduced into the culture system to a final concentration of 0.2 mM, inducing the expression of the target protein. Subsequently, when the bacterial OD_600_ reached approximately 1.0, the cultivation temperature was lowered to 32°C to promote the expression of *sfGFP*. The biomass values were measured using a gain setting of 1 on the BioLector XT micro bioreactor.

### Fed-Batch Fermentation in a Bioreactor

Recombinant *E. coli* strains stored at -80°C were streak-inoculated onto LB solid plates and incubated at 37°C for 12 h to revive the cultures. Individual colonies were then selected and inoculated into 10 ml of LB liquid medium, followed by incubation at 37°C with shaking at 200 rpm for 12-14 h to obtain a seed culture. The seed culture was subsequently transferred into a 250 ml sterile conical flask containing 30 ml of LB liquid medium, utilizing an inoculation ratio of 1:10, and incubated at 37°C with shaking at 220 rpm for 16 h to establish a secondary seed culture. This secondary seed culture was then used to prepare a tertiary seed culture by inoculating into two 2 L sterile conical flasks, each containing 200 ml of MR fermentation medium as previously described [23], again with an inoculation ratio of 1:10. The culture was incubated at a constant temperature of 37°C with shaking at 220 rpm for 12-15 h. Finally, the tertiary seed culture was inoculated into a 5-L fermenter (Baoxing, China) containing 2 L of MR01 medium at the same inoculation ratio of 1:10. The pH of the fermentation broth was maintained at 7.0 through the addition of 30% phosphoric acid and ammonia, while the dissolved oxygen level was consistently kept between 30% and 40% by adjusting the agitation rate and aeration (3 l/min). During the initial phase of fermentation (0-12 h), the temperature was maintained at 37°C. Upon reaching a cell density of OD_600_ =10, heterologous vitamin B_12_ pathway genes were induced by lowering the temperature to 32°C. A sterile glycerol solution (40%, w/v) was automatically added when glycerol levels were depleted, ensuring residual glycerol levels remained below 5 g/l. Samples were collected periodically at specified intervals to assess cell growth, residual glycerol concentration, and the production of the target product, vitamin B_12_.

### Analytical Methods

The quantification of vitamin B_12_ in the fermentation broth was conducted using high-performance liquid chromatography (HPLC). The procedure involved the following steps: 20 ml of fermentation broth was diluted with double-distilled water (ddH_2_O) to achieve a final volume of 2 ml, resulting in a 10-fold dilution. Subsequently, 200 ml of an 8% sodium nitrite solution and 200 ml of glacial acetic acid were added to the diluted solution. After thorough mixing, the solution was autoclaved at 100°C for 30 min. to release the bound form of vitamin B_12_. Following the reaction, the sample was allowed to cool naturally to room temperature and then centrifuged at 5,000 rpm for 5 min. to separate the supernatant. The supernatant was filtered through a 0.22 mm membrane to eliminate impurities. Finally, the filtered solution was analyzed using an Agilent 1260 series HPLC system equipped with a C18 column (Agilent, 5 µm × 250 × 4.6 mm) and a UV detector set at 320 nm. The mobile phase A was composed of ddH_2_O, while mobile phase B consisted of pure methanol. The elution gradient was set to a ratio of A to B at 3:7, and the analysis was conducted at a temperature of 35°C with a flow rate of 0.8 ml/min. The quantification of vitamin B_12_ was based on a standard curve.

### Statistical Analysis

Significant variations among the experimental factors were assessed using one-way ANOVA, followed by Tukey's multiple comparisons test, with a significance level set at *p* < 0.05. All analyses were conducted using GraphPad Prism software (Version 10.1.0).

## Results and Discussion

### Design of T7*lac* and *lac*-Derived Promoters Based on a Thermal Switch

The T7*lac* promoter, a robust construct derived from the T7 phage and lactose operon promoters [17], is extensively utilized in recombinant protein expression systems. In the absence of IPTG, the LacI protein binds to the *lacO* site of the T7*lac* promoter, effectively blocking the transcription of the downstream gene. Conversely, in the presence of IPTG, LacI is released upon binding IPTG, allowing for the transcription of the downstream gene. This regulatory mechanism is also applicable to other promoters, such as the tac and lacUV5 promoters, which contain *lacO* and are similarly regulated by LacI.

**Fig. 1:**
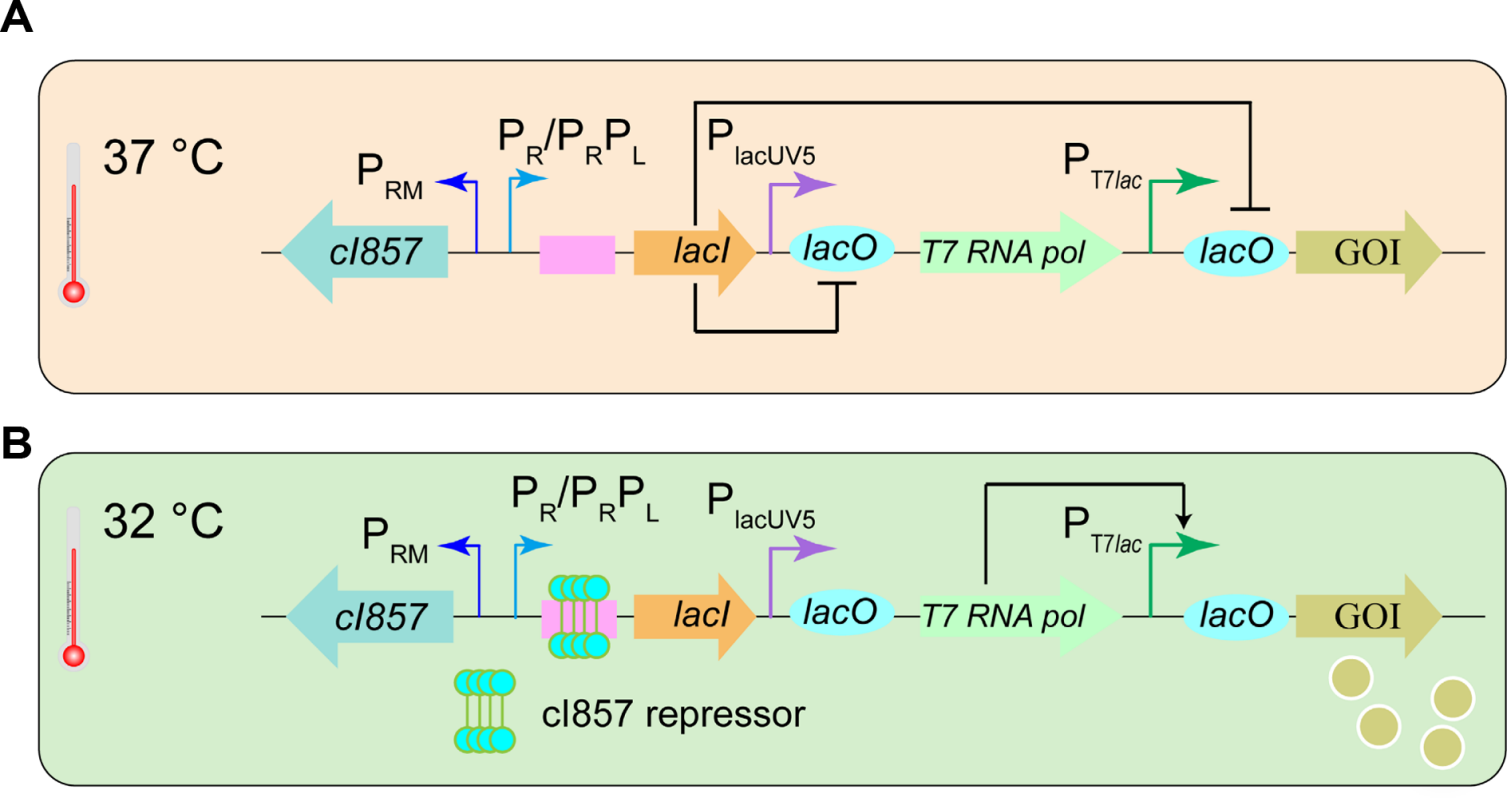
An overview of the thermal switch mechanism used to control gene expression. (**A**) At elevated temperatures (37°C), the P_R_, or tandem P_R_, P_L_ promoter becomes dissociated from the inactive temperature-sensitive repressor CI857, allowing LacI to bind to the *lacO* site of the lacUV5 and T7*lac* promoters. As a result, the gene of interest is not expressed. (**B**) Conversely, at lower temperatures (32°C), transcription of the P_R_, or tandem P_R_, P_L_ promoter is inhibited by the temperaturesensitive repressor CI857. Consequently, the lacUV5 and T7*lac* promoters are liberated from LacI, which enables the expression of the gene of interest (**GOI**).

Cell lysis was observed during the fermentation of recombinant vitamin B_12_-producing strains, which was attributed to the toxicity of IPTG (Fig. S1) [5]. To eliminate the reliance on IPTG, we aimed to design a thermal switch to regulate genes driven by the T7*lac* promoter. Previous studies have presented a thermo-regulated T7 expression system; however, the operational temperature range of 39°C to 37°C is considered too high for the effective expression of heterologous genes. Consequently, we positioned the *lacI* gene under the control of the P_R_, or tandem P_R_ and P_L_ promoters, which are regulated by the temperature-sensitive repressor cI857 (Fig. 1). At elevated temperatures (37°C), which corresponds to the optimal growth conditions for *E. coli*, the cI857 repressor is inactive, leading to the transcription of the *lacI* gene as shown in Fig. 1A. Consequently, the transcription of downstream T7*lac* genes, including gene 1, which encodes T7 RNA polymerase, is effectively inhibited. For instance, under these conditions, the target gene *sfGFP*, which is regulated by the T7*lac* promoter, remains untranscribed, as do the genes related to the vitamin B_12_ pathway that are governed by the T7*lac*, tac, and lacUV5 promoters. Upon shifting to a lower temperature of 32°C, which is conducive to the expression of heterologous proteins, the cI857 repressor becomes active, and transcription of the *lacI* gene ceases (Fig. 1B). This change facilitates the transcription of *sfGFP* and the vitamin B_12_ pathway genes. By modulating *lacI* gene expression via the P_R_, or P_R_P_L_ promoters, we enable temperature-based regulation of all promoters that fall under the control of LacI.

### Characterization of the Thermoregulated Promoters

**Fig. 2:**
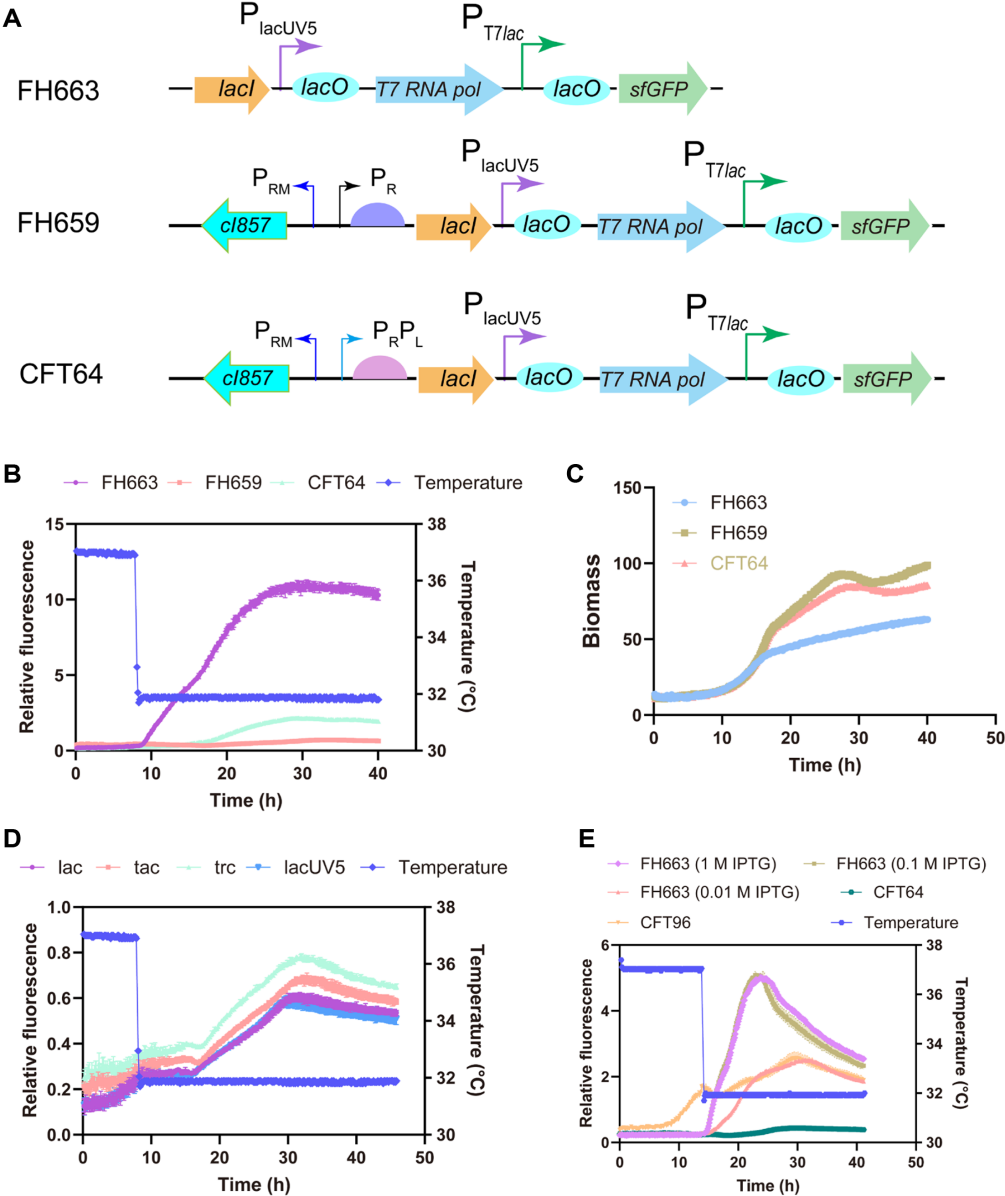
Characterization of the thermal switch using the *sfGFP* reporter. (**A**) Characteristics of FH663 controlled by IPTG, along with strains regulated by the thermal switch. The *lacI* gene is regulated by the P_R_ promoter (FH659) or the tandem P_R_, P_L_ promoter (CFT64), respectively. (**B**) Fluorescence curves for FH663, FH659, and CFT64 at varying temperatures. The *sfGFP* in strain FH663 was induced by 1 mM IPTG when the temperature increased to 32°C. Conversely, the *sfGFP* in strains FH659 and CFT64 was induced at lower temperatures before shifting to 32°C. (**C**) Growth curves for FH663, FH659, and CFT64 under the same conditions as described in (**B**). Biomass values were recorded with a gain setting of 1 on the BioLector XT micro bioreactor. (**D**) Fluorescence curves for strains with the *sfGFP* gene regulated by lac, tac, trc, and lacUV5 promoters under the control of the thermal switch. (**E**) A comparison of the fluorescence curves of FH663, CFT64, and CFT96 (with down-regulation of *lacI* under the control of the P_R_P_L_ promoter). The FH663 strain was induced with varying concentrations of IPTG when the temperature shifted to 32°C, while the CFT64 and CFT96 strains were induced by the lower temperature (32°C). Error bars indicate standard deviations from triplicate (Fig. 2B-2D) or quadruplicate (Fig. 2E) biological replicates.

We employed the SfGFP as a reporter to assess the thermoregulated expression system. Two chassis cells, FH478 and CFT81, were engineered by substituting the promoter of the *lacI* gene in the chromosome with the P_R_ and P_R_P_L_ promoters, respectively. Subsequently, the *sfGFP* gene, regulated by the T7*lac* promoter, was integrated into the *hsdR* locus of both FH478 and CFT81, resulting in the creation of FH659 and CFT64, respectively (Fig. 2A).

Microbioreactor cultivations of the strains FH659, CFT64, and the control strain FH663 were utilized to monitor green fluorescent protein (GFP) fluorescence and biomass. Following a temperature downshift to 32°C, the fluorescence levels of FH659 and CFT64 gradually increased, peaking at 33 h and 29 h, respectively (Fig. 2B), thereby indicating effective operation of the thermo-regulated expression system. The relative fluorescence of FH659 and CFT64 was lower than that of the control strain FH663, which was induced with 1 mM IPTG, with the maximum relative fluorescence of CFT64 reaching 19.69% of the control. However, the biomasses of the strains utilizing the thermo-regulated expression system were significantly greater than that of the control at the 16-h mark (Fig. 2C), suggesting that the thermo-regulated expression system mitigates the negative effects associated with IPTG induction. Furthermore, the efficacy of the thermo-regulated expression system was maintained when the *sfGFP* gene was regulated by the *lac*, tac, trc, and lacUV5 promoters containing *lac* operator (*lacO*) (Fig. 2D). The versatility of such a system could enable researchers to manipulate gene expression across various pathways and applications, including metabolic engineering, protein production, and synthetic circuit design. Given that the dynamic range of the thermo-regulated expression system is narrower than that of the IPTG-regulated expression system, we aimed to enhance the dynamic range. We hypothesized that the ratio of LacI to cI857 significantly impacts the expression of thermo-regulated target genes. To test this hypothesis, we modified the RBS of CFT64 to create a strain designated CFT96 with a weaker RBS. Notably, the relative fluorescence of CFT96, when *lacI* expression was downregulated, reached levels comparable to the control induced by 0.01 mM IPTG (Fig. 2E). However, the decrease in *lacI* expression led to undesired leaky expression of *sfGFP*, as evidenced by the increasing trend of relative fluorescence at 37°C.

### Biosynthesis of Vitamin B_12_ Using Pathway Genes Controlled by the Thermal Switch

While the dynamic range of the thermo-regulated expression system is narrower than that of the IPTG-regulated expression system, it remains promising due to its ability to mitigate the negative effects associated with IPTG, reduce costs, and enhance biomass production. To optimize the production of the target compound, the transcriptional levels of genes must be maintained within appropriate ranges rather than at maximal levels [5, 24]. In this study, we employed the thermo-regulated expression system using two plasmid-free recombinant *E. coli* strains, specifically JH03 [3] and JPTB38. However, we were only able to successfully obtain the strains B58 and B54, which feature *lacI* driven by the P_R_ promoter. Attempts to develop strains with *lacI* driven by the P_R_P_L_ promoter were unsuccessful, likely due to the significant burden of heterologous protein expression imposed by that configuration. In both strains B58 and B54, five modules comprising 28 heterologous genes involved in the vitamin B_12_ biosynthetic pathway were driven by T7*lac*, tac, and trc promoters, thereby placing these genes under the control of the thermal switch (Fig. S2).

**Fig. 3:**
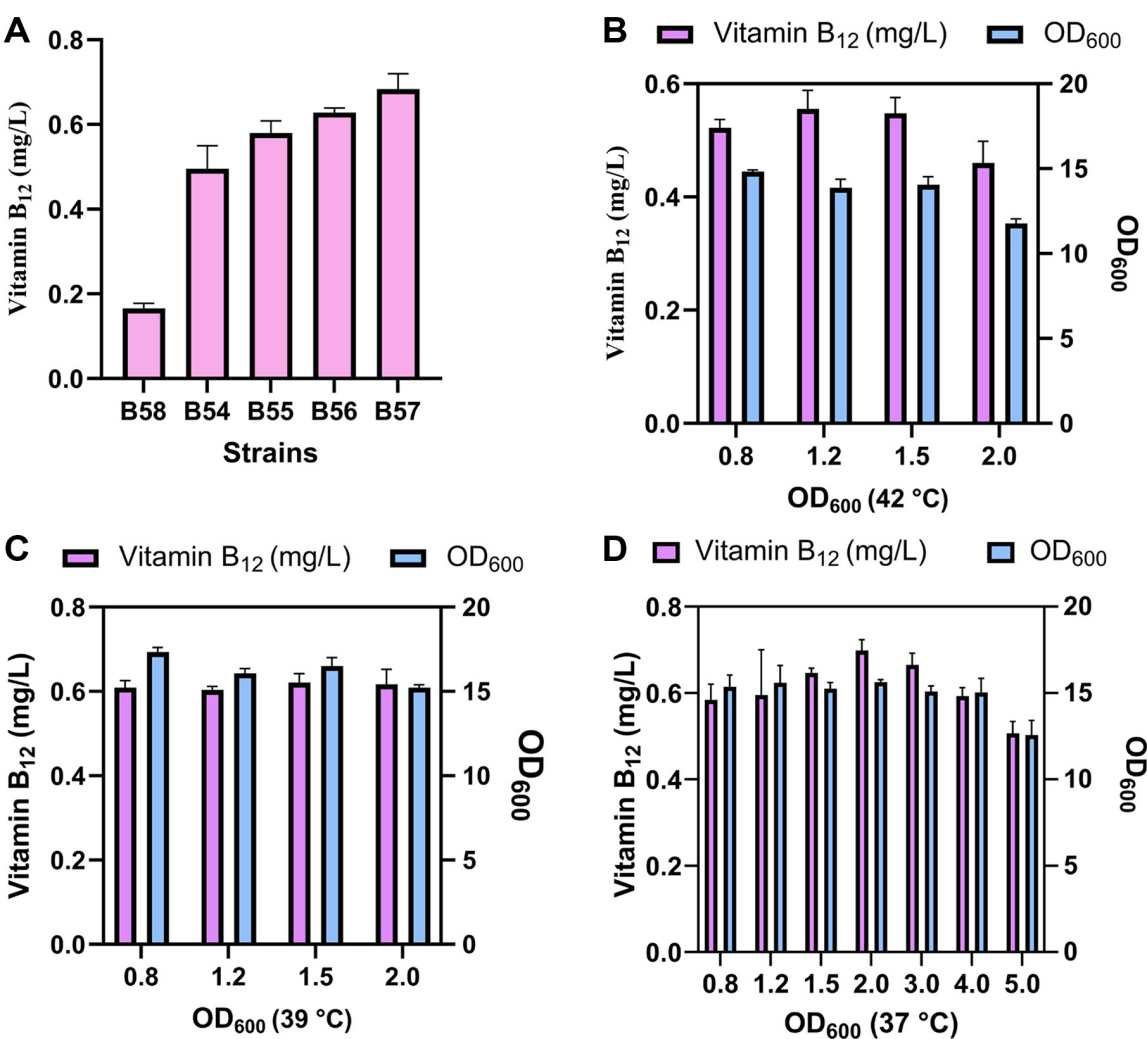
Optimization of the thermal switch for vitamin B_12_ production. (**A**) The vitamin B_12_ titers of recombinant *E. coli* strains B58 and B54, which were derived from different host organisms. Strains B55, B56, and B57 were engineered by incorporating *ssrA* degradation tags, specifically LVA, LAA, and ASV tags, at the C terminus of the *lacI* gene of the B54 strain. The B57 strain was initially cultivated at temperatures of 42°C (**B**) 39°C (**C**) and 37°C (**D**). Temperature shifts to 32°C were executed once the OD_600_ reached the corresponding values indicated on each x-coordinate. Error bars indicate standard deviations from triplicate biological replicates.

The thermal switch was subsequently assessed in these two strains. They were initially cultured at 37°C, and upon reaching an OD_600_ of 0.8-1.0-which corresponds to the IPTG-inducing time point—the temperature was lowered to 32°C to facilitate vitamin B_12_ production. The strains B58 and B54 yielded production levels of 0.166 mg/l and 0.495 mg/l of vitamin B_12_, respectively (Fig. 3A). Following this, we sought to enhance the vitamin B_12_ titer in the B54 strain. Given that reducing *lacI* expression at the translational level resulted in leaky expression, we decided to decrease *lacI* expression at the post-translational level instead. Bacteria can recognize and degrade proteins tagged with the *ssrA* degradation tag—an 11-residue peptide-using proteases such as ClpXP and ClpAP [25]. We introduced three *ssrA* degradation tags with varying strengths (LVA, LAA, and ASV) co-translationally to the C-terminus of the *lacI* gene in the B54 strain, resulting in strains B55, B56, and B57. The vitamin B_12_ titers in all three strains carrying *lacI*-*ssrA* tags were increased (Fig. 3A). Notably, the B57 strain, which carried the ASV tag (the weakest of the three), exhibited the highest increase in vitamin B_12_ titer, rising by 37.96%.

The implementation of the thermal switch segregated the fermentation process into two distinct phases: a high-temperature phase and a low-temperature phase. Three critical parameters-growing temperature, producing temperature, and switching time point-significantly influenced vitamin B_12_ production. Given that 32°C is recognized as the optimal temperature for vitamin B_12_ production [19], we focused on optimizing the growing temperature and switching timepoint to enhance production outcomes. The B57 strain was initially cultivated at various temperatures (42°C, 39°C, and 37°C) and subsequently transitioned to 32°C at different time points, as indicated by OD_600_ readings. At an initial temperature of 42°C, vitamin B_12_ titers did not exhibit significant variation when switching timepoint (OD_600_) increased from 0.8 to 1.5 (*p* > 0.05) (Fig. 3B). Conversely, the vitamin B_12_ titers at an initial temperature of 39°C remained relatively stable across different timepoints (Fig. 3C). Overall, the optimal initial temperature for vitamin B_12_ production was determined to be 37°C based on the highest vitamin B_12_ titers observed in each group (*p* < 0.05), which corresponds with the optimal growth temperature of 37°C. In conclusion, the optimal initial temperature for vitamin B_12_ production (approximately 0.7 mg/l) was determined to be 37°C, as it resulted in the highest vitamin B_12_ titers across all groups (*p* < 0.05) despite the highest biomass being recorded at 39°C (*p* < 0.05). The optimal switch time points across OD_600_ values ranging from 0.8 to 4 did not reveal any statistically significant differences (*p* > 0.05) (Fig. 3D). The highest vitamin B_12_ titer was achieved at a switch time point of OD_600_ = 2, although this result was not statistically significant. Notably, the lowest biomass was statistically observed at the highest OD_600_ across all groups during temperature shifts (*p* < 0.01), with the exception of the group with a switch point of OD_600_ = 2 at an initial temperature of 39°C (p = 0.0512), likely attributable to the burden of heterologous proteins expressed at lower temperatures.

### Fed-Batch Fermentation for Vitamin B_12_ in a 5-L Bioreactor

**Fig. 4:**
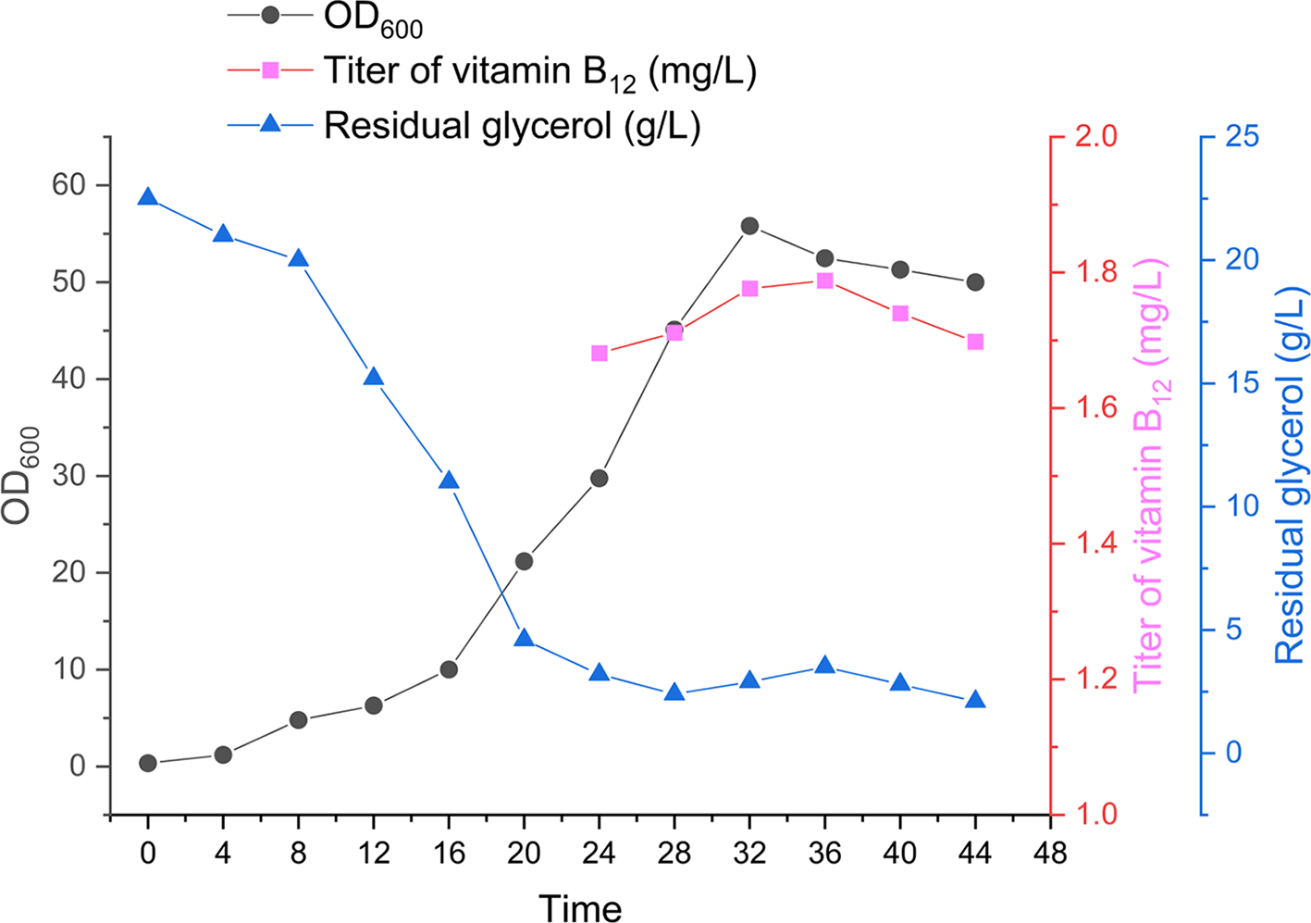
Fed-batch production of vitamin B_12_ utilizing B57 in a 5-L bioreactor. The time courses of vitamin B_12_ titer, OD_600_, and residual glycerol concentrations are illustrated.

To assess the impact of the thermal switch on scaled-up vitamin B_12_ production, fed-batch fermentation of strain B57 was performed in a 5-L bioreactor. Consistent with the shake-flask fermentation, the strain was initially cultivated at 37°C and subsequently transitioned to 32°C upon reaching the mid-logarithmic phase after 12 h. Strain B57 experienced a lag phase of up to 12 h at the onset of fermentation, followed by the logarithmic phase, during which it achieved a maximum biomass of 55.8 approximately 32 h into the process (see Fig. 4). vitamin B_12_ production paralleled biomass accumulation, gradually increasing from 24 h to 36 h, ultimately reaching a maximum titer of 1.79 mg/l at 36 h. Following this peak, production declined in tandem with the deterioration of the strain.

In conclusion, this study successfully demonstrates the design and implementation of a thermal switch based on the phage l Promoter and *lacI*, enabling regulation of gene expression in metabolic pathway engineering without the need for IPTG. The most effective approach for producing vitamin B_12_ involves initially culturing at 37°C and then transferring to 32°C once the optical density at 600 nm reaches 2. Furthermore, the introduction of an ASV degradation tag at the C-terminus of *lacI* has been shown to enhance the vitamin B_12_ titer.

## Supplemental Materials

Supplementary data for this paper are available on-line only at http://jmb.or.kr.
