# Supplementary material for: Production of Vitamin B12 in Escherichia coli Using a Thermal Switch to Control Pathway Genes
Source: J Microbiol Biotechnol. 2025 Apr 11;35:e2412068. doi: 10.4014/jmb.2412.12068 (PMC12010068; doi:10.4014/jmb.2412.12068)
Supplement: Supplementary file 1 [file jmb-35-e2412068-supple.pdf]

J. Microbiol. Biotechnol. 2025. 35: e2412068

https://doi.org/10.4014/jmb.2412.12068

Supplementary Tables and Figures

Table S1. Strains and plasmids used in this study.

Strains and plasmids	Description	Reference or source
FH224	MG1655 (DE3)△*endA*	This study
FH478	FH224△P_{lacI} promoter::P_R promoter	This study
FH663	FH224△hsdR::P_{T7lac}-*sfGFP*	This study
FH659	FH478△hsdR::P_{T7lac}-*sfGFP*	This study
CFT64	FH663△P_{lacI} promoter::P_RP_L promoter	This study
CFT96	FH663△P_{lacI} promoter-RBS::P_RP_L promoter-weak RBS	This study
CFT76	FH478△hsdR::P_{lac}-*sfGFP*	This study
CFT77	FH478△hsdR::P_{tac}-*sfGFP*	This study
CFT78	FH478△hsdR::P_{trc}-*sfGFP*	This study
CFT79	FH478△hsdR::P_{lacUV5}-*sfGFP*	This study
FH517	A strain producing co(II)byrinic acid a,c-diamide	Unpublished
JH03	FH517 △ yjiV::P_{T7lac}-PacbiBEC-P_{T7lac}-*SmcobRAP*	[1]
JPTB38	FH517 △ araD::P_{T7lac}-cbiBXD-P_{T7lac}-*BmcobRAP*	Unpublished
B58	JH03△P_{lacI} promoter::P_R promoter	This study
B54	JPTB38△P_{lacI} promoter::P_R promoter	This study
B55	B54 lacI::LVA tag	This study
B56	B54 lacI::LAA tag	This study
B57	B54 lacI::ASV tag	This study
pCas9-hsdR-sfGFP	Cas9 plasmid for inserting the P_{T7lac}-*sfGFP* cassette	This study
pCas9-hsdR-lac-sfGFP	Cas9 plasmid for inserting the P_{lac}-*sfGFP* cassette	This study
pCas9-hsdR-tac-sfGFP	Cas9 plasmid for inserting the P_{tac}-*sfGFP* cassette	This study

pCas9-hsdR-trc-sfGFP	Cas9 plasmid for inserting the P_{trc}-*sfGFP* cassette	This study
pCas9-hsdR-lacUV5-sfGFP	Cas9 plasmid for inserting the P_{lacUV5}-*sfGFP* cassette	This study
pCas9-PR-lacI	Cas9 plasmid for replacing the *lacI* pomoter to P_R	This study
pCas9-PRPL	Cas9 plasmid for replacing the *lacI* pomoter to P_RP_L	This study
pCas9-RBS-lacI	Cas9 plasmid for down-regulating the *lacI*	This study
pCas9-lacI-LVA tag	Cas9 plasmid for adding of LVA tag to the C-terminal of *lacI*	This study
pCas9-lacI-LAA tag	Cas9 plasmid for adding of LAA tag to the C-terminal of *lacI*	This study
pCas9-lacI-ASV tag	Cas9 plasmid for adding of ASV tag to the C-terminal of *lacI*	This study

Tables S2. Primers used in this study.

Primer	Sequence (5'→3')
For the construction of pCas9-PR-lacI and pCas9-PRPL	
Pcas9-1-F-Pr-lacI	agatcttagcCGGTATGGCATGATAGCGCCgttttagagctagaaatagcaag
Pcas9-1-R-Pr-lacI	GGCGGTTTTCAGAAGCGGCAacaggcccatggattct
Pcas9-2-F-Pr-lacI	ACCAGGATGCCATTGCTGTGtgaatggaagcttggattctc
Pcas9-2-R-Pr-lacI	gctctaaaacGGCGCTATCATGCCATACCGgctaagatctgactccataac
Pr-lacI-up-F	cgaagaatccatgggcctgtTGCCGCTTCTGAAAACCG
Pr-lacI-up-R	taacaattgagcaagaatctTCAACGTAAATGCATGCCG
Pr-F	GCGGCATGCATTTACGTTGAAGATTCTTGCTCAATTGTTATCAGC
Pr-R	TATAACGTTACTGGTTTCACACAACCTCCTTAGTACATGCAAC
Pr-lacI-dn-F	gcatgtactaaggaggttgtGTGAAACCAGTAACGTTATACG
Pr-lacI-dn-R	agaatccaagcttccattcaCACAGCAATGGCATCCTG
pcas9-Pr-lacI-F	GTGAAACCAGTAACGTTATACG
pcas9-Pr-lacI-R	ACAACCTCCTTAGTACATGCAAC
PL-F	gcatgtactaaggaggttgtATGGAACAACGCATAACCCTG
PL-R	TATAACGTTACTGGTTTCACGAATTCCTCCTTAATTTTTAACCAATGC
For the construction of pCas9-hsdR-lac-sfGFP	
lac-hsdR-F	GCTCGTATGTTGTGTGGAAGGAATTGTGAGCGGATAACAATTCC
lac-hsdR-R	CGGAAGCATAAAGTGTAAAGTTGAGTAACAGACCAAGATGTTTCG
For the construction of pCas9-hsdR-tac-sfGFP	
tac-hsdR-F	CTCGTATAATGTGTGGAAGGAATTGTGAGCGGATAACAATTCC
tac-hsdR-R	CCGATGATTAATTGTCAAGTTGAGTAACAGACCAAGATGTTTCG
For the construction of pCas9-hsdR-trc-sfGFP	
trc-hsdR-F	gctcgtataatgTGTGGAAGGAATTGTGAGCGGATAACAATTCC
trc-hsdR-R	cggatgattaattgtcaaGTTGAGTAACAGACCAAGATGTTTCG
For the construction of pCas9-hsdR-lacUV5-sfGFP	
lacUV5-hsdR-F	gctcgtataatgTGTGGAAGGAATTGTGAGCGGATAACAATTCC
lacUV5-hsdR-R	cggaagcataaagtgtaaaGTTGAGTAACAGACCAAGATGTTTCG
For the construction of pCas9-RBS-lacI	
lacI-up-PR-F	CGAAGAATCCATGGGCCTG
PL-RBS2-R	CTGGTTTCACTTGCTTCCCGCTACTACTAGCccaatgcttcgtttcgtatcac

lacI-RBS2-F	aagcattggGCTAGTAGTAGCGGGAAGCAAGTGAAACCAGTAACGTTATACG
Pr-lacI-dn-R	agaatccaagcttccattcaCACAGCAATGGCATCCTG
lacI-RBS-SOE-R	AGAATCCAAGCTTCCATTCACA

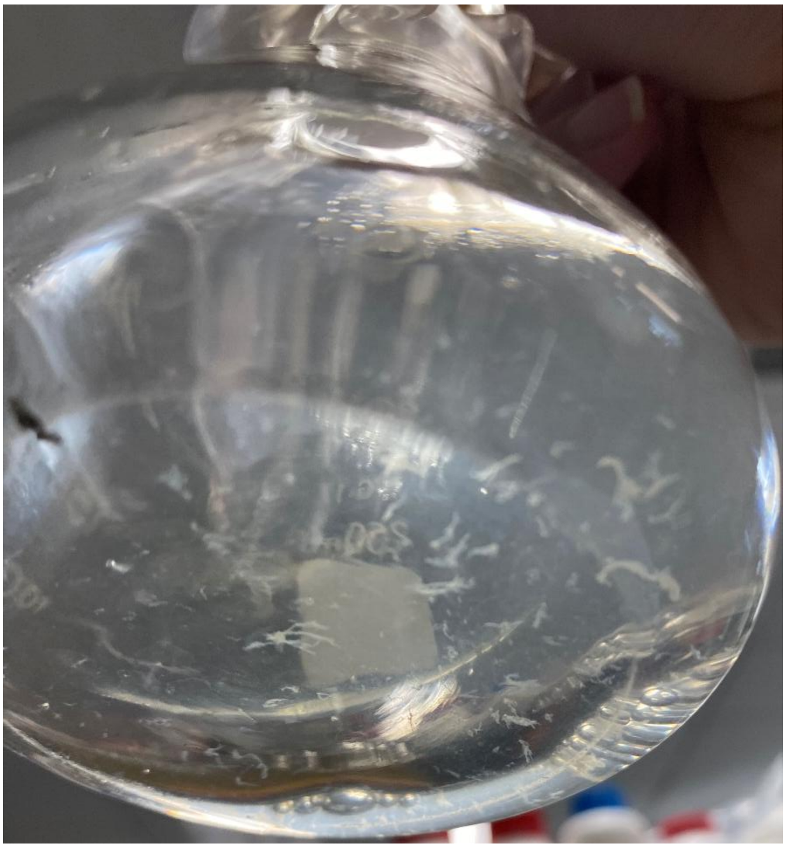

Fig. S1. Cell lysis of due to IPTG toxicity during the fermentation of recombinant vitamin B_{12}-producing strain.

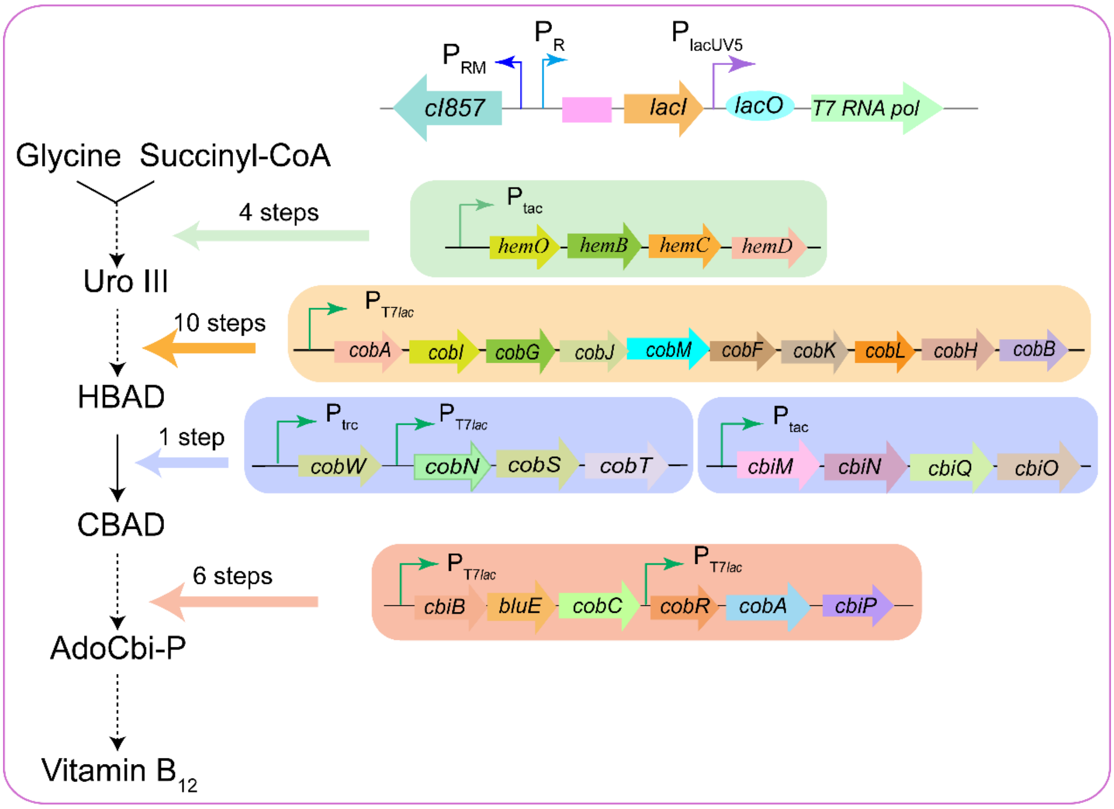

Fig. S2. Modules of the B58 strain regulated by the thermal switch. Uro III, uroporphyrinogen III; HBAD, hydrogenobyrinic acid a,c-diamide; CBAD, co(II)byrinic acid a,c-diamide; AdoCbi-P, adenosylcobinamide-phosphate.

[1] Fang, H., Zhao, J., Zhao, X., Dong, N., et al., Standardized Iterative Genome Editing Method for *Escherichia coli* Based on CRISPR-Cas9. *ACS Synth. Biol.* 2024, *13*, 613–623.
